# Pathological Mechanisms of Skin Homing T Cells in Atopic Dermatitis

**DOI:** 10.1097/WOX.0b013e3181d675f8

**Published:** 2010-03-15

**Authors:** Marta Ferran, Luis F Santamaria-Babi

**Affiliations:** 1 Department of Dermatology, Hospital del Mar, IMAS-IMIM, Barcelona, Spain; 2 Institute for Research in Biomedicine, University of Barcelona, Barcelona, Spain

**Keywords:** homing, CLA, skin, atopic eczema, T cell

## Abstract

Skin infiltration of circulating memory T cells with cutaneous tropism is considered one of the pathologic mechanisms in atopic dermatitis (AD). Skin-seeking circulating T lymphocytes can be identified by their expression of the cutaneous lymphocyte-associated antigen on the cell surface. Recent studies have contributed useful new information about the function and recirculation properties of those cells in AD patients. This review integrates the latest findings on peripheral cutaneous lymphocyte-associated antigen memory T cells in AD and highlights the relevance of this cell type and its importance to our understanding of the pathologic mechanisms of AD.

## Introduction

Atopic dermatitits (AD) is the most common childhood skin disease, with a steadily increasing prevalence over recent decades. Characterized by chronic and relapsing flares of intrinsic or extrinsic eczema, AD is considered to be part of the atopic state, which includes asthma, allergic rhinitis, allergic conjunctivitis, and food allergies. AD normally precedes all of these conditions [1]. The exact etiopathogenic mechanisms are unknown but genetics, immunologic, and environmental factors have been involved, and defects in skin barrier function [2]. Our understanding of AD pathogenesis expanded with the recent identification of its association with loss-of-function mutations in filaggrin, an epidermal protein important for cutaneous barrier integrity. These mutations produce a heritable epithelial barrier dysfunction that allows allergens and microbes easy access; this may cause a polarized T_H_2 lymphocyte response, which results in eczema [2]. However, it has also been suggested that T_H_2 cytokines could influence barrier function, modulating the expression of filaggrin or other structural proteins and peptides important for microbial barrier function [3]. Without taking into account their primary or secondary role in the defective barrier function in AD, it is known that T cells play an important role in the pathologic mechanism of AD. The fact that drugs acting on immune-mediated mechanisms are the most effective treatments for AD at the moment (corticosteroids and immuno-modulators such as tacrolimus or pimecrolimus) supports their pathogenic role.

## Circulating memory CLA^+ ^T lymphocytes

Circulating memory cutaneous lymphocyte-associated antigen (CLA)^+ ^T cells participate in the inflammatory reaction produced during the initiation of AD lesion [4-6]. Recent studies, reviewed below, support the role of CLA^+ ^T cells as one of the pathologic mechanisms in AD, and the reduced innate immune response and abnormal skin barrier function [2].

CLA, a surface glycoprotein expressed in 15% of peripheral blood lymphocytes, acts as a skin homing molecule [7,8]. CLA is expressed in > 90% of infiltrating T cells present in inflamed skin, but is found in < 20% of T lymphocytes in other inflamed sites such as gut, joints, or lungs [9].

CLA is a carbohydrate posttranslational modification of the ubiquitously expressed p-selectin glycoprotein ligand-1 (PSGL-1) by the enzymatic activity of fucosyltransferase VII (FucT-VII) [10]. Interleukin (IL)-12, which drives naive T cells into T_H_1 cell differentiation, is also the best characterized mediator responsible for conferring skin homing to both T_H_1 and T_H_2 cells, by inducing FucT-VII [5]. Superantigens may have a role in the induction of CLA expression in certain disorders such as AD [11]. At present, it is believed that tissue-specific homing receptors are imprinted on T cells in tissue-draining lymph nodes. This is best demonstrated by homing markers for the gut and skin by gut-draining lymph nodes and skin-draining lymph nodes, respectively. Dendritic cells and soluble factors are believed to mediate this immunologic imprinting [12-14].

Significant experimental and clinical results support the concept that leukocyte migration to tissues under inflammatory conditions is not a random process but rather is orchestrated through molecules such as adhesion molecules and chemokines [7]. The CLA antigen is part of a complex multistep molecular interaction between circulating lymphocytes and cutaneous vascular endothelium that takes place during lymphocyte migration to the skin. Under inflammatory conditions, proinflammatory mediators like IL-1 and tumor necrosis factor (TNF)-*α *up-regulate the expression of adhesion molecules on the surface of endothelial cells. In inflammation, the interactions between CLA/E-selectin, Very Late Antigen-4(VLA-4)/Vascular Cell Adhesion Molecule-1(VCAM-1) and Lymphocyte Function-Associated Antigen-1(LFA-1)/Inter-Cellular Adhesion Molecule 1 (ICAM-1) are required in the transendothelial migration of CLA^+ ^T cells [15].

## Clinical relevance of cutaneous chemokines that attract circulating cla+ t cells to inflamed skin in ad

Besides the array of adhesion molecules implicated in T cell transendothelial migrations, circulating CLA^+ ^T cells require chemo-attractant stimuli, provided by specific chemo-kines, to reach the inflamed skin. It is known that the chemokine receptors CCR4 and CCR10 are highly expressed on circulating CLA^+ ^T cells [4,16,17]. Thymus and activation-regulated chemokine (TARC/CCL17) and macrophage-derived chemokine (MDC/CCL22) are the chemokines ligands specific for CCR4, whereas the cutaneous T cell-attracting chemokine (CTACK/CCL27) is specific for CCR10 [18]. The production of MDC, TARC, and CTACK is well established in AD lesions, [19] and recent studies have correlated the expression of these chemokines/chemokine receptors involved in CLA^+ ^T cell chemotaxis with clinical severity of AD. In addition, cutaneous CCL18 is associated with AD, which mediates skin homing of human memory T cells [20,21].

TARC is expressed by keratinocytes, vascular endothelial cells, T cells, and dendritic cells [22]. Intradermal injection of TARC into human skin grafted on severe combined immunodeficient (SCID) mice reconstituted with peripheral blood mononuclear cells results, 24 hours later, in a cutaneous recruitment of human memory CD4(+) cells, monocytes, and basophiles, but also murine eosinophils. In SCID mice reconstituted with polarized Th-1 or Th-2 cells, intradermal injection of TARC resulted in the recruitment of IL-4(+) Th-2 cells but not of IFN-γ(+) Th-1 cells [4,23]. In AD patients, TARC/CCL17 levels are significantly higher than those of healthy control subjects and patients with psoriasis. Increased serum TARC levels in AD patients decreases after treatment, in accordance with improvement in clinical symptoms. Furthermore, an association between serum levels and AD disease activity has been found [24].

MDC is produced by macrophages and dendritic cells upon activation and its serum levels are higher in AD patients than in healthy controls and psoriasis patients [25]. Increases in serum MDC levels are greater in severe AD patients than in moderate or mild AD cohorts, decrease after treatment, and significantly correlates with the Scoring AD (SCORAD) index [25] and CCL18 [21]. Interestingly, the CCR4 receptor, which binds both MDC and TARC, is preferentially expressed on CD4^+ ^T cells from AD patients. The expression of CCR4 is more prominent in severe AD subjects and decreases when disease activity improves [26].

Finally, CTACK is produced by basal keratinocytes; [17] the serum levels of this chemokine in patients with AD are significantly higher than in healthy control subjects, and correlate with SCORAD [27]. A differential gene expression study comparing peripheral blood CD4^+ ^T cells from AD patients and healthy controls identified increased expression of some genes in AD patients. Some of those genes were associated to tissue homing, such as CCR10, the specific receptor for CCL27 [28]. The FucT VII^+ ^CLA^+ ^T_H_2 subset, which has the most potent capacity to bind E-selectin, increased dramatically in the blood of AD patients. This increase was related to the severity of clinical symptoms [29].

All together, these studies suggest a correlation between AD severity and serum levels of CLA^+ ^T cell-attracting chemokines such as MDC, TARC, and CTACK, which can be reduced with treatment. Because inflamed skin is the source of these mediators, one possible chemokine mechanism is to attract circulating CLA[+] T cells to the inflammatory focus. This is not a mere infiltration process of T cells into the skin. When all the pieces of information about CLA^+ ^T cells are put together, a clearer picture emerges of the relevance of chemokines-attracting skin-homing T cells to disease severity.

## Circulating CLA^+ ^T cells contain subsets of t cells that respond to cutaneous allergens

The CLA antigen is not expressed on naive CD45RA^+ ^cells, but rather on a subset of antigen-experienced memory effector T cells [30]. Memory effector T cells constitute a subpopulation of lymphocytes able to produce a wide range of cytokines that are involved in inflammation. It is well established that only CLA^+ ^T cells from AD patients, but not asthmatics or healthy controls, preferentially respond to cutaneous allergens such as *Dermatophagoides pteronyssinus *in AD [31]. Thus, in AD the CLA^+ ^T cell-attracting chemokines are involved in the migration of circulating allergen-experienced T cells to skin. One possible hypothesis is that under such circumstances increased trafficking of allergen-specific T cells from blood to skin takes place, resulting in a correlation between serum levels of chemokines and disease severity. Furthermore, skin-infiltrating CLA^+ ^T cells not only respond to cutaneous allergens in AD, but are also exposed to microorganisms that can cause cutaneous infection, such as *Staphylococcus aureus *(*S. aureus*). Cutaneous infections in AD are a common event that exacerbates the disease [32]. Patients with active AD present a higher percentage of cells positive for the T cell receptor (TCR) V*β*2 and V*β*5.1 segments in the CLA^+ ^but not in the CLA^- ^subset [33]. Thus, chemokines-attracting CLA^+ ^T cells will contribute to the infiltration mechanism of allergen-specific and *S. aureus *superantigen-responsive T cells that could boost allergic inflammation in AD.

The use of biologic treatments that are specifically directed toward relevant molecules involved in lymphocyte trafficking offers opportunities to understand the pathologic mechanism in AD. This is the case of efalizumab, an anti-LFA-1 antibody. LFA-1, an adhesion molecule of the integrin family, binds to ICAM-1 on endothelial cells and mediates leukocyte extravasation. Furthermore, LFA-1 is involved in CLA^+ ^T cell dependent transendothelial migration [34]. Lesions improved in AD patients treated with efalizumab; at the same time, patients presented a marked increase in circulating memory CD4^+ ^T cells that express the CLA antigen [15]. One possible explanation for this effect would be a continuous recirculation of CLA^+ ^T cells between skin lesions and peripheral blood: when LFA-1 is blocked, CLA^+ ^T cells from the skin accumulate in circulation [34]. Although these CLA^+ ^T cells are effector memory T cells, it appears that circulating T cells from AD patients present decreased expression of apoptosis-related genes compared with the same subtype in healthy individuals, indicating a prolonged survival [34]. In support of the recirculation hypothesis, some activation markers (HLA-DR, CD25, and CD69) are expressed in CLA^+ ^T cells present in peripheral blood [31,35]. In addition, it has been shown that circulating CLA^+ ^T cells in AD spontaneously produce T_H_2 cytokines (IL-4, IL-5, and IL-13) without need of TCR activation, [31,36] suggesting that those cells have been previously activated.

Recently, the implication of CLA^+ ^T cell function in AD has also expanded to itching. IL-31 is a newly identified cytokine that when overexpressed in mice induces pruritus, [37] providing a new link between CLA^+ ^T cells and itching in AD patients. IL-31 is produced by activated T lymphocytes, and preferentially by T_H_2 cells. In vivo application of Staphylococcal superantigen to skin can rapidly induce IL-31 in atopic individuals and its receptor presents the most abundant expression in dorsal root ganglia, the anatomic location of the cell bodies of cutaneous sensory neurons [38]. In addition, IL-31 receptor is also expressed by keratinocytes and macrophages. Human keratinocytes activated by IL-31 produce different types of chemokines, such as TARC (CCL17) and MDC (CCL22) [37]. IL-31 is exclusively produced by circulating CLA^+ ^T cells, and superantigen enterotoxin B activation of peripheral blood lymphocytes induced production of IL-31 [38]. Recent findings suggest IL-31 antibody as a new potential therapeutic approach for pruritus in AD and other pruritic diseases. Finally, other neurogenic mechanisms involve CLA^+ ^T cells in AD. Calcitonin gene-related peptide has been shown to directly activate CLA^+ ^T cells and increase IL-13 production in AD patients, [39] and mental stress in AD increased the number of CLA^+ ^T cells in circulation [40].

## Circulation of CLA^+ ^T cells in ad

In summary, circulating CLA^+ ^T cells with potential to recognize allergens related to AD and bacterial infections are present in peripheral blood. T lymphocytes with skin tropism are attracted into the cutaneous inflammatory focus through adhesion molecules expressing endothelial cells (E-selectin, VCAM-1, and ICAM-1) and the production of chemokines MDC, TARC, CTACK, and CCL18. There is a relationship between clinical severity and the presence of CLA^+ ^T cell-attracting chemokines in the serum, suggesting that continuous T cell infiltration may take place during cutaneous inflammation.

**Figure 1:**
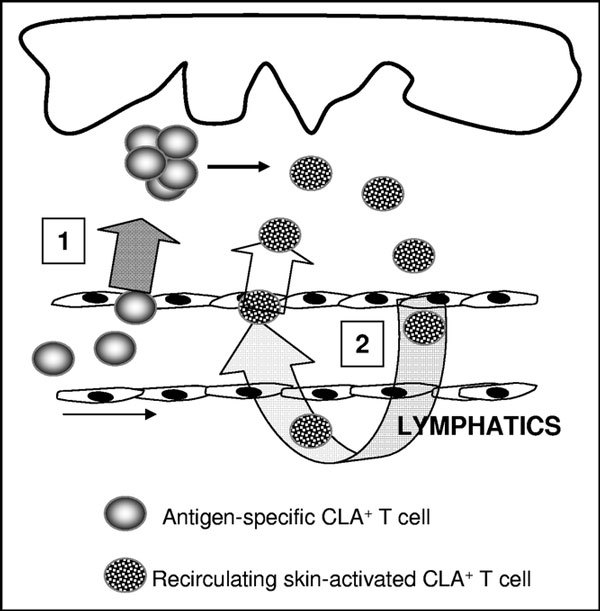
**During cutaneous inflammation in AD, CLA^+ ^T cell-selective chemokines are produced that allow continuous migration of skin-reactive CLA^+ ^T cells to inflamed lesions (step 1)**. Once in the inflamed skin, CLA^+ ^T cells are activated and a portion of the memory T cells return to the blood through the thoracic duct (step 2). CLA^+ ^T cells present in the blood present features of recent activation and spontaneous production of cytokines and can migrate back to inflamed skin.

In acute AD, circulating CLA^+ ^T cells preferentially express T_H_2 cytokines (IL-4, IL-5, and IL-13) in comparison to controls or CLA-negative cells. In addition, CLA^+ ^T cells produce IL-31 upon activation, which links this skin-homing population to itching in AD. As shown in Figure 1, it can be hypothesized that CLA^+ ^T cells recirculate between blood and inflamed lesions in AD, similar to the dynamics of memory T cell recall response in peripheral tissue [8].

Peripheral CLA^+ ^T cells express activation markers and reduced levels of apoptosis-related genes in AD patients, and spontaneously produce cytokines also detected in skin lesions. An anti-LFA-1 antibody that has been shown to interfere with T cell memory trafficking into skin may induce CLA^+ ^T cells lymphocytosis. Recirculation between skin and blood could persist for as long as the attracting gradient and adhesion molecules specific for CLA^+ ^are present in the lesions. Once a lesion improves, a pool of circulating CLA^+ ^T cells with skin-homing potential that can participate in future allergic inflammation is present in the blood of AD patients, by virtue of the memory pool generated during inflammation.
